# Effect of nerve growth factor on sperm quality in asthenozoosprmic men during cryopreservation

**DOI:** 10.1186/s12958-016-0163-z

**Published:** 2016-05-27

**Authors:** Sara Saeednia, Maryam Shabani Nashtaei, Hossein Bahadoran, Ashraf Aleyasin, Fardin Amidi

**Affiliations:** School of medicine, Shahroud University of Medical Sciences, Shahroud, Iran; Department of Anatomy, School of medicine, Tehran University of Medical Sciences, Tehran, Iran; Department of Anatomy, School of Medicine, Baqiyatallah University of Medical Sciences, Tehran, Iran; Department of Infertility, Shariati Hospital, Tehran University of Medical Sciences, Tehran, Iran

**Keywords:** Nerve growth factor, Cryopreservation, Nitric oxide, DNA fragmentation, Human spermatozoa

## Abstract

**Background:**

Although routinely used in assisted reproductive technology, human sperm cryopreservation is not an entirely successful procedure. This study determined the effect of nerve growth factor (NGF) supplementation of cryopreservation medium on post-thaw viability, motility, intracellular nitric oxide (NO) concentration, and DNA fragmentation of human spermatozoa in asthenozoospermic men.

**Methods:**

Semen samples were collected from 25 asthenozoosprmic men and divided into the following groups (*n* = 5/group): fresh semen (control); frozen-thawed semen without treatment; frozen-thawed semen with NGF treatment (0.5, 1, and 5 ng/ml). Prior to dividing the asthenozoospermic samples, 200 μl of each sample was collected for NGF content assessment by ELISA and then compared with normozoospermic semen samples (25 normozoospermic men). Sperm motility and viability were assessed according to WHO criteria. Furthermore, intracellular nitric oxide and DNA fragmentation were evaluated by Flow Cytometry.

**Results:**

NGF content was significantly higher in normozoospermic compared with asthenozoospermic men. Cryopreservation of asthenozoospermic semen samples significantly decreased sperm viability and motility, and increased intracellular nitric oxide concentration and DNA damage (p < 0.01). In asthenozoospermic frozen–thawed samples treated with 0.5 ng/ml exogenous NGF, we observed a significantly increased viability, motility, and decreased DNA fragmentation (p < 0.05), but intracellular nitric oxide concentration was not reduced. The other high doses (1 and 5 ng/ml) had no significant effect on the variables.

**Conclusion:**

Supplementation with exogenous NGF could have partial and limited protective effect during cryopreservation of human spermatozoa but further research is needed to evaluate the possible clinical applications.

## Background

Cryopreservation is a useful strategy in long term storage of sperm cells and conservation of male fertility [1]. However, during cryopreservation spermatozoa are affected by physical and chemical stresses that cause damages in membrane lipid composition, motility, viability, acrosome status, and fertilization capacity of spermatozoa [2, 3]. In brief, cryopreservation results in the elevation of lipid peroxidation rate in the plasma membrane which can induce an excessive increase in reactive oxygen species (ROS) concentration and can eventually lead to oxidative stress. This oxidative stress can adversely affect DNA integrity [4]. In view of paternal genetic contribution, mammalian chromatin structure integrity is one of the vital parameters for normal fetus development and healthy offspring [5].

It is also recognized that cryopreservation can induce sperm cells to lose their antioxidant defense systems [6]. Moreover, in poor semen samples, the concentration of ROS is unusually high while the effects of endogenous antioxidants are often diminished [7, 8].

In order to improve and maintain sperm quality during cryopreservation, the application of antioxidants has recently been extensively studied to decrease the deleterious effects of ROS [7–10]. NGF, an antioxidant supplement, is a member of neurotrophins which has reported to play an important role in the male reproductive system and sperm function [11–15]. NGF stimulates sperm motility [12, 16, 17], viability [13] and facilitates sperm cell acrosome reactions [12], and also play an important role in the proliferation and differentiation of leydig cells and testosterone production [18].

Furthermore, intracellular nitric oxide (NO), a free radical, is another factor that physiologically regulates spermatozoa function, and pathophysiology of male reproductive system [19, 20]. The role of NO in multiple signal transduction pathways of male germ cells like capacitation and acrosomal reaction is well documented [21]. The motility of mouse [22], hamster [23], and human [24, 25] spermatozoa, and also the zona pellucida-binding ability of human spermatozoa were improved by low concentrations of NO. On the contrary, maturity of human spermatozoa was correlated with active NO production by NO synthase and inactivated apoptosis signaling, indicating a rather anti-apoptotic effect of NO [21].

Since there is limited evidence for the cryoprotective effects of NGF on human spermatozoa especially in asthenozoospermic samples, we investigated the effects of the addition of NGF to the freezing and thawing extender on viability, motility, intracellular nitric oxide concentration, and DNA fragmentation, and also assessment of semen NGF content in asthenozoospermic men.

## Methods

### Collection and preparation of samples

This study was approved by the Ethics Committee of Baqiyatallah Medical University and was performed in accordance with national and international guidelines. All subjects were informed with respect to this study and a written consent to participate in the study was obtained, as well as informed consent to publish their clinical data.

Semen samples were collected from 25 normozoospermic and 25 asthenozoospermic donors referred to Shariati hospital via masturbation following 3–5 days of sexual abstinence. After liquefaction at 37°^C^ and 5 % CO_2_, a basic seminal analysis was carried out according to the 5^th^ edition of World Health Organization guidelines [26]. Prior to dividing the asthenozoospermic semen samples, 200 μl of each sample was collected for semen NGF content assessment and then compared with normozoospermic semen samples (25 normozoospermic men). The asthenozoospermic (*n* = 25) samples were further divided into the following subgroups (*n* = 5/group): Fresh semen (control); frozen-thawed semen samples without treatment; frozen-thawed semen samples with NGF treatment (final concentration: 0.5, 1, and 5 ng/ml).

### Freezing and thawing process

In the fresh semen sample (control) groups, all parameters were assessed freshly. In the other groups, after the addition of equal volume (1:1) of pre-warmed sperm freezing medium (Vitrolife, Sweden), the mixture was swirled lightly and cryotubes were kept at room temperature for 10 min. Afterward, the cryotubes were suspended in the liquid nitrogen vapor at −80°^C^ (15–30 cm above the level of liquid nitrogen) for 20–30 min. Then, they were plunged into liquid nitrogen and stored for two weeks. The samples were then thawed at room temperature for 5 min, and incubated at 37°^C^ for 20 min. The freezing medium was removed by centrifugation at 1000 rpm for 5 min and the appropriate medium, according to the protocol which will be mentioned for each assessment, was added.

### Determination of NGF content of semen

NGF assessment was evaluated using an enzyme-linked immunosorbent assay (ELISA) kit (abcam ab99986, USA). This assay employs an antibody specific for human NGF coated on a 96-well plate. All reagents, samples and standards were prepared as instructed. 100 μl of each of a standard or a sample was transferred into each well and then incubated for 2.5 h at room temperature. 100 μl of prepared biotin antibody was added to each well and incubated, again, for 1 h at room temperature. The wells were then washed to remove any unbound biotinylated antibody. HRP conjugated streptavidin was pipetted to the wells and incubated for 45 min at room temperature. The wells were washed again and 100 μl of TMB substrate solution was added to the wells. The intensity of the color that formed in the wells, was in proportion to the amount of bound NGF. Stop Solution was added which changed the color from blue to yellow, and the intensity of the color was measured at 450 nm.

### Assessment of sperm viability and motility

Sperm viability was evaluated using Eosin/Nigrosin stain. Staining was performed by mixing 20 μl of semen with 20 μl of Eosine (1 %). Afterwards, 20 μl of Nigrosin (10 %) was added to each solution. A smear was made on a glass slide and allowed to air-dry. Unstained (intact) and red (with disrupted membranes) spermatozoa were counted under oil immersion light microscopy at × 1000 magnification. Vitality was quantified by counting a minimum of 200 spermatozoa on each slide, and the proportion of membrane-intact spermatozoa was expressed as a percentage of total cell number.

Sperm motility parameters were analyzed according to World Health Organization guidelines [26]. A 10 μl drop of gently mixed semen was put on a microscope slide and covered with coverslip and assessed using a phase contrast microscope at 4000 magnification in multiple views.

### Intracellular nitric oxide (NO) detection

Briefly, for NO measurements each sample was loaded with DAF-2/DA (4,5 diaminofluorescein- 2/diacetate) and incubated in the dark (120 min, 37°^C^) before being analyzed by fluorescence-activated cell sorter (FACS) [27]. Excitation wavelength (488 nm) and emission wavelength (530 nm) were used in the single-cell level, and data were analyzed using Cellquest^TM^ version 3.3 software (Becton Dickinson, San Jose, CA, USA). The mean fluorescence intensity of the analyzed sperm cells was ascertained after gating the cell population by 90° and forward-angle light scatter to exclude debris and aggregates. The final gated populations usually consisted of 8000–12000 sperm cells. Fluorescence in these cells was recorded on a frequency histogram by logarithmic amplifiers.

### Evaluation of sperm DNA fragmentation

In situ cell death detection kit, fluorescein (Roche, 11684795910, Germany) was applied to detect sperm DNA fragmentation according to manufacturer’s instruction. Using this kit, apoptosis at the single cell was quantified. Briefly, 3 × 10^6^ cells were washed twice with phosphate-buffered saline (PBS; pH 7.4), then fixed with 200 μL of freshly prepared 4 % paraformaldehyde for 1 h at room temperature in the dark. Afterwards, sperm cells were washed with PBS and permeabilized with 0.1 % triton X-100 in 0.1 % sodium citrate for 15 min on ice. After washing with PBS, the labelling reaction was performed by incubating sperm cells with 50 μl of TUNEL reaction mixture (Tdt enzyme and fluorescein isothiocyanate conjugate [FITC]-labeled nucleotides) for 1 h at 37°^C^ in a humidified atmosphere in the dark. Finally, the cells were washed twice with PBS and analyzed using flow cytometry. Assessment of a negative control (sample without the addition of Tdt enzyme) and a positive control (sample treated with DNase I (3 U/ml, Invitrogen) for 10 min at room temperature to produce DNA strand breaks) by TUNEL assay is necessary. Finally, samples were washed twice with PBS and analyzed with flow cytometry. For each semen sample, sperm DNA fragmentation was determined before and after cryostorage [10].

### Statistical analysis

Data are expressed as the mean ± SEM. *T*-test was used to analyze NGF concentration in normozoospermic and asthenozoospermic semen samples. Viability, motility, intracellular nitric oxide, and DNA fragmentation were analyzed by one-way ANOVA in order to assess the effect of NGF in the different groups. When variances in the Levene test were statistically significant, Dunnett T3 for multiple comparison post hoc tests was used. If variances in the Levene test were not statistically significant, Tukey HSD for multiple comparison post hoc tests was used. Differences were regarded as statistically significant at *p* < 0.05.

## Results

### NGF content in normozoospermic and asthenozoosprmic men

NGF concentration was measured with ELISA kits. Concentrations were calculated from respective standard curves and expressed as pictogram per milligram of entire protein. The NGF content (mean ± SEM) was significantly higher in normozoospermic (1160 ± 94.71) compared with asthenozoospermic (563 ± 44.58) samples (*P*-value <0.05).

### Effect of exogenous NGF on viability

**Fig. 1:**
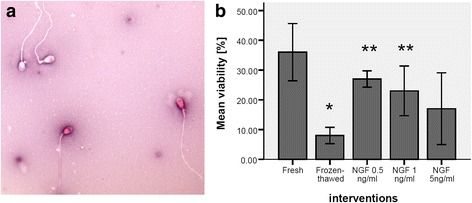
Effect of nerve growth factor on sperm viability in asthenozoospermic men (**a**). Human spermatozoa staining with Eosin/Nigrosin dye assessed by oil immersion light microscopy at × 1000 magnification. Live spermatozoa appeared white whilst dead spermatozoa with disrupted membranes have taken up the Eosin stain and appeared red (**b**). *P*-values <0.05 were considered significant.*: Significant difference vs. fresh group (*P*-value <0.05). **: Significant difference vs. frozen-thawed group (*P*-value <0.05). Error bars: ±1 SE

In asthenozoospermic men (*n* = 25) viability of frozen-thawed group (8 ± 1.22) was significantly reduced compared with fresh group (36 ± 4.30) (*P*-value <0.00). The addition of NGF in freezing medium at 0.5 ng/ml (27 ± 1.22) and 1 ng/ml (23 ± 3.74) significantly increased viability vs. frozen-thawed group (8 ± 1.22) (*P*-value <0.05). But viability in frozen-thawed samples with exogenous NGF at 5 ng/ml (17 ± 5.38) had no significant difference compared with frozen-thawed group (8 ± 1.22) (*P*-value > 0.02) (Fig. 1a).

### Effect of exogenous NGF on motility

The percentage of sperm motility in all groups was evaluated and has been summarized in Table 1. In asthenozoospermic men (*n* = 25), percentage of total motility of sperm in fresh group (45 ± 1.97) had a significant increase vs. frozen-thawed group (10 ± 1.57) (*P* < 0.001), whereas percentage of immotile sperm in the frozen-thawed group increased significantly compared with fresh group (*P* < 0.001). Treatment of frozen–thawed samples with exogenous NGF at 0.5 ng/ml increased the percentage of total motility (27 ± 3.81) and also showed a significantly decrease in the percentage of immotile sperm vs. frozen–thawed group (*P* < 0.001) (Table 1).

**Table 1:** Effect of nerve growth factor (NFG) on sperm motility in asthenozoospermic men

	Groups	Total motility (Mean ± SEM)
	Fresh	1.97 ± 45
	Frozen – thawed	1.57 ± 10
Asthenozoospermic men	NGF 0.5 ng/ml	3.81 ± 27
	NGF 1 ng/ml	3.66 ± 17
	NGF 5 ng/ml	2.16 ± 14

### Effect of exogenous NGF on nitric oxide

**Fig. 2:**
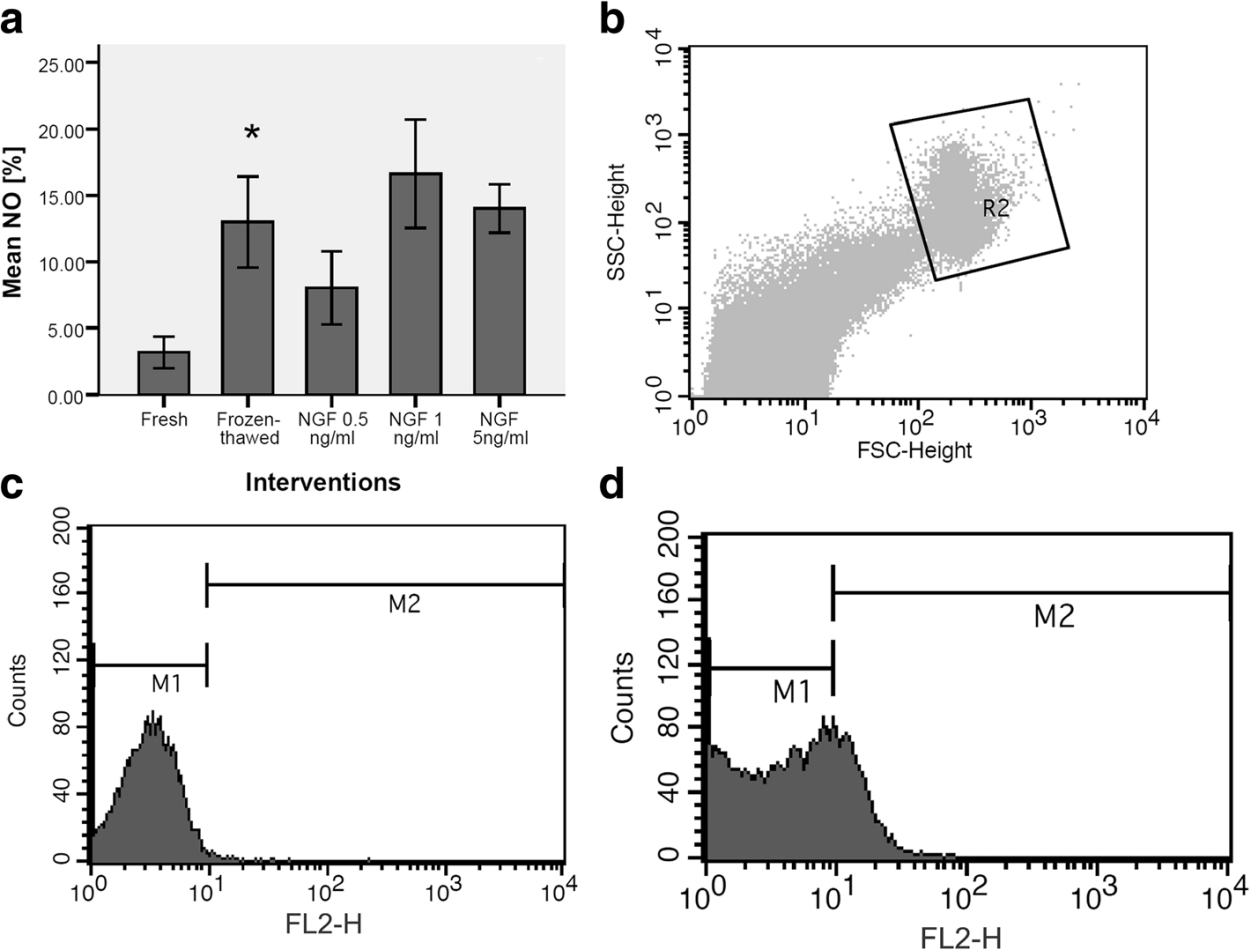
Effect of nerve growth factor on sperm nitric oxide content in normozoospermic men (**a**). Dot plot represents total acquired events and final gated population (R2) of spermatozoa (**b**). Histogram of negative control without incubation with baseline 4, 5-diaminofluorescein-2/diacetate (DAF-2/DA) fluorescence (M1) (**c**). Histogram of one of semen samples, incubated with DAF-2/DA fluorescence. The population producing nitric oxide corresponds to M2 region of the histograms (**d**). *P*-values <0.05 were considered significant.*: Significant difference vs. fresh group (*P*-value <0.05). **: Significant difference vs. frozen-thawed group (*P*-value <0.05). Error bars: ±1 SE

In asthenozoospermic men, NO content of frozen-thawed group (13 ± 1.53) showed significant elevation vs. fresh group (3.18 ± 0.52) (*P*-value <0.01). The addition of NGF in freezing medium at all concentrations could not cause any change in NO content of samples vs frozen-thawed group (Fig. 2a, b, c, and d).

### Effect of exogenous NGF on apoptosis

**Fig. 3:**
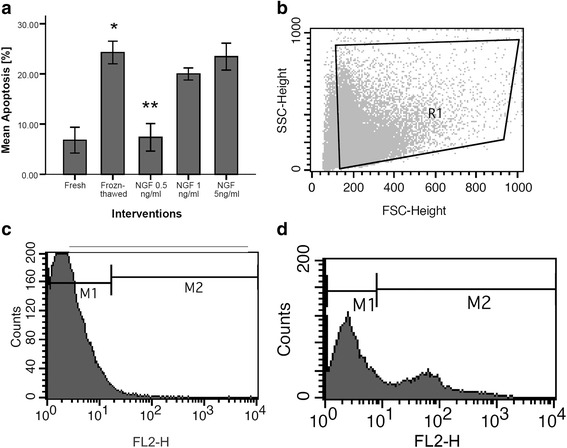
Effect of nerve growth factor on sperm DNA fragmentation in asthenozoospermic men (**a**). Dot plot represents total acquired events and final gated (R1) population of spermatozoa (**b**). Histogram of negative control (without deoxynucleotidyl transferase enzyme) with 1.35 % TUNEL positive cells (M1) (**c**). Histogram of one of semen samples (stained by dUTP-FITC) with 9.1 % TUNEL positive cells (M2) (**d**). *P*-values <0.05 were considered significant.*: Significant difference vs. fresh group (*P*-value <0.05). **: Significant difference vs. frozen-thawed group (*P*-value <0.05). Error bars: ±1 SE

In asthenozoospermic men, DNA damage rate of frozen-thawed group (24.26 ± 1.00) was significantly increased vs. fresh group (6.80 ± 1.14) (*P*-value <0.01). The addition of NGF at 0.5 ng/ml (7.37 ± 1.22) in freezing medium showed significant difference in DNA fragmentation compared with frozen-thawed group (24.26 ± 1.00) (*P* < 0.01). But NGF at 1 ng/ml (19.99 ± 0.530) and 5 ng/ml (23.44 ± 1.19) had no significant difference vs. frozen-thawed group (24.26 ± 1.00) (*P*-value >0.1) (Fig. 3a). Dot plot and histogram of semen samples that were analyzed by flow cytometry are shown in Fig. 3b, c, and d.

## Discussion

Freezing and thawing cycle is associated with oxidative stress affecting those processes required for successful in vivo and in vitro fertilization of the oocyte. Production of ROS and alteration in antioxidant defense systems have been reported to be the significant contributing factors [4, 6, 28, 29]. Therefore various antioxidant compounds along with cryo-media have been implemented to minimize sperm cryoinjury and improve post-thaw semen parameters [7–10].

In the current study, we treated freeze-thawed spermatozoa with NGF at different concentrations to determine whether this member of the neurotrophin protein family could improve the post-thaw viability, motility, intracellular nitric oxide, and DNA integrity of spermatozoa in asthenozoospermic men.

Nerve growth factor is probably the most extensively studied member of neurotrophins which its function is mediated through tyrosine kinase receptor A (TrkA) [30]. Many studies have shown its presence in all stages of germinal cells. Moreover, the impact of NGF on the reproductive systems [11, 13, 15] and functions of spermatozoa was recently assessed in animal model researches indicating its role in promoting the formation and development of testis, the spermatozoon differentiation, maturation [31, 32], viability [13], and motility [12], and also DNA integrity in human hippocampus [33]. The first evidence of a measured NGF concentration in human elucidated that oligoasthenozoospermic men, compared with asthenozoospermic and fertile men, have lower NGF concentrations in their seminal plasma. TrkA mRNA levels are also significantly lower in spermatozoa samples from oligoasthenozoospermic men than in those from asthenozoospermic and fertile men [34]. Similar to the study above, NGF content was also found to be increased in normozoospermic samples in our previous study [17].

Cryopreservation promotes ROS production and thus can induce lipid peroxidation leading to decreased sperm viability and activity of mitochondria whilst inducing sperm apoptosis [2, 35]. Previous studies demonstrated that addition of exogenous NGF to the incubation medium had significant effects on bovine [13] and human [17] sperm cell viability, and also exhibits trophic activity in the rescue of sertoli cell viability mediated by the Trk receptor protein [36]. In addition, the positive effect of NGF addition (0.5 and 1 ng/ml) to the freezing extender on human sperm viability in asthenozoospermic men has been demonstrated according to our results.

Motility is one of the most important features that play a crucial role in fertilizing ability of spermatozoa [37]. The freezing-thawing procedure results in DNA fragmentation, and can also suppress glycolysis pathway and ATP production leading to a reduction in sperm motility [38]. However, sperm cell motility significantly increases following using different antioxidants as freezing extender [8–10]. It has been demonstrated that NGF which exhibits an antioxidant activity can promote the parameters of sperm motility in a time- and dose-dependent manner [12, 16]. Furthermore, we previously reported that exogenous NGF as cryoprotectant improved post-thaw motility of frozen sperm in normozoospermic men [17]. According to this study, post-thawed asthenozoospermic samples along with NGF 0.5 ng/ml were also more motile. In this regard, these findings may promote the clinical application of NGF in ARTs.

As far as sperm functions are concerned, NO is synthesized through the oxidation of L-arginine to L-citruline by three isoforms of reduced NADPH-dependent NO synthases (NOS), and plays an important role in capacitation and acrosomal reaction [21, 39–41]. NO stimulates human sperm motility via soluble guanylate cyclase activation, and subsequent cGMP synthesis and cGMP-dependent protein kinases activation [25]. NO is produced both in fresh and frozen-thawed stallion spermatozoa, and its production increases after cryopreservation [42], as in cryopreserved human spermatozoa of asthenozoospermic samples. Moreover, our preceding report regarding normozoospermic cases disclosed that, in compare with untreated frozen-thawed samples, addition of 0.5 ng/ml NGF to the freezing medium followed by lower NO concentration, and consequently higher viability and motility [17].

Some studies reported that cryopreservation resulted in DNA damage of human spermatozoa which is induced by ROS formation [4, 8, 43]. In recent years, different pro-survival factors such as antioxidants have been added to cryopreservation medium to decrease the effect of ROS on DNA integrity [7, 10, 37]. As a result, maintenance of DNA integrity of frozen-thawed spermatozoa is one of the important indicators to assess the effectiveness of cryopreservation [7]. NGF is one supplementation which its effect has been determined on different cells. Nguyen et al. have shown that NGF prevents staurosporine -induced apoptotic morphology and caspase-3 activity in hippocampus, by upregulating phosphorylation of the tropomyosin receptor kinase (Trk) receptor [33]. It has also been found that endothelial cells and skeletal myocytes are not only protected from apoptosis, by the use of exogenous NGF, but also induce neovascularization in murine ischemic limb muscles and diabetic skin wounds [44, 45]. In dopaminergic cells, NGF prevents rotenone-induced neurotoxic apoptosis through the activation of the PI3-kinase pathway [46]. Furthermore, NGF may play an essential role in cell survival as an antiapoptotic factor in non-neuronal osteoblastic cells and inhibiting the apoptosis of rat peritoneal mast cells [47].

In our previous report, supplementation of the extender with 0.5 ng/ml NGF lead to decrease DNA fragmentation compared with untreated frozen–thawed samples [17]. Although in the present study we showed that the effect of NGF (0.5 ng/ml) lead to improve DNA integrity of asthenozoospermic samples, it seems protective effect of NGF in normozoospermic cases is more noticeable. It is possible that cryodamage to sperm DNA integrity of asthenozoospermic samples is much higher.

Furthermore, in spite of the protective effect of NGF at 0.5 ng/ml on viability, motility, and DNA fragmentation, the other high doses (1 and 5 ng/ml) had no significant effects. It is feasible that these higher concentrations exerted a negative effect through an extreme scavenging and antioxidant activity or interfering with intracellular pathways during vital steps of the cryopreservation procedure.

However, only a partial aspect of the cryoprotective effect of NGF has been analyzed and further examinations will be needed to assess the clinical application of NGF for improving post-thawed human sperm parameters and investigating the possible interactions between NGF and the other semen parameters to explain detailed mechanisms of its functions.

## Conclusion

In conclusion, a significant improvement in asthenozoospermic human sperm viability, motility, and apoptosis was observed when NGF was added in the freezing media. This study suggests that NGF supplementation can be profitable for successful freezing in asthenozoospermic men due to their lower NGF content.

## Abbreviations

DAF-2/DA, 4,5 diaminofluorescein- 2/diacetate; ELISA, enzyme-linked immunosorbent assay; FACS, fluorescence-activated cell sorter; NGF, nerve growth factor; NO, nitric oxide; NOS, NO synthases; ROS, reactive oxygen species; TrkA, tyrosine kinase receptor A.
